# Anisotropic Conductivity and Mechanical Strength Enhancements in Gel Polymer Electrolyte Films by Hot Pressing

**DOI:** 10.3390/ma18081751

**Published:** 2025-04-11

**Authors:** Zhifan Fang, Hao Zhang, Shuangjun Chen

**Affiliations:** 1 College of Material Science & Engineering, Nanjing Tech University, Nanjing 210009, China; 202261103021@njtech.edu.cn (Z.F.); 15950590506@163.com (H.Z.); 2 Jiangsu Collaborative Innovation Center for Advanced Inorganic Function Composites, Nanjing Tech University, Nanjing 210009, China

**Keywords:** gel polymer electrolyte, poly(vinylidene fluoride), ionic liquid, recrystallization, ion conductivity, mechanical strength

## Abstract

Gel polymer electrolyte (GPE) with a polymer matrix swollen in liquid electrolytes offers several advantages over conventional liquid electrolytes, including no leakage, lightweight properties, and high reliability. While poly(vinylidene fluoride-co-hexafluoropropylene) (PVDF-HFP)-based GPEs show promise for lithium-ion batteries, their practical application is hindered by the intrinsic trade-off between ionic conductivity and mechanical robustness in conventional PVDF systems. Typical strategies relying on excessive plasticizers (e.g., ionic liquids) compromise mechanical integrity. Here, we propose a novel hot-pressing-induced recrystallization strategy to synergistically enhance both anisotropic ionic conductivity and puncture strength in PVDF-based GPE films. By blending PVDF with controlled amounts of 1-hexyl-3-methylimidazolium chloride ([HMIM]Cl), followed by solution casting and hot pressing, we achieve microstructural reorganization that dramatically improves through-thickness ion transport and mechanical performance. Crucially, hot-pressed PVDF with only 25 wt% [HMIM]Cl exhibits a 12.5-fold increase in ionic conductivity (reaching 4.7 × 10^−4^ S/cm) compared to its solution-cast counterparts. Remarkably, this formulation surpasses the conductivity of PVDF-HFP composites with a higher [HMIM]Cl content (35 wt%, 1.7 × 10^−4^ S/cm), demonstrating performance optimization of anisotropic conductivity. What is more, the mechanical strength of the piercing strength perpendicular to the GPE film after hot pressing increased by 42% compared to the solution-cast film. This work establishes a scalable processing route to break the conductivity–strength dichotomy in GPEs, offering critical insights for designing high-performance polymer electrolytes.

## 1. Introduction

Rechargeable lithium-ion batteries (LIBs) with high energy density and long cyclic life have been widely used in various types of power and energy storage devices [1,2,3,4]. The rising demand for high-performance batteries necessitates a reevaluation of the conventional properties of liquid electrolytes, which are prone to flammability and leakage [3,5,6,7]. Gel polymer electrolyte (GPE) with a polymer matrix saturated in liquid electrolytes offers several advantages over conventional liquid electrolytes, including no leakage, lightweight properties, and high reliability [2,8,9,10]. Within the diverse array of gel polymer electrolyte (GPE) systems, poly(vinylidene fluoride) (PVDF) copolymers demonstrate notable attributes, including significant hydrophobicity, exceptional chemical resistance, and enhanced thermal and mechanical properties [11]. These attributes make PVDF copolymers the ideal host polymer for the development of high-quality gel polymer electrolytes [12,13,14,15]. Nonetheless, the quest for enhanced ionic conductivity frequently leads to a deterioration in the mechanical properties of PVDF-based gel polymer electrolytes (GPE), consequently constraining their potential applications [2,4,16,17].

The addition of inorganic nanofillers to enhance the conductivity of PVDF-based electrolytes is a common strategy [18,19,20,21]. Yi et al. successfully developed and synthesized a gel polymer electrolyte (GPE) composed of a poly(vinylidene-fluoride-co-hexafluoropropylene) (PVDF-HFP)/poly(methyl methacrylate) (PMMA) matrix GPE containing bis(trifluoromethane)sulfonimide lithium salt (LiTFSI) and spherical zirconium dioxide (ZrO_2_) nanoparticles. This was achieved through a straightforward one-step solution-casting method. It has been found that the PVDF-HFP/PMMA-ZrO_2_-6% (PPZ-6%) electrolyte film obtained possesses an excellent tensile strength of 37.7 MPa. Moreover, the PPZ-6% GPE has a high ionic conductivity of 1.46 × 10^−3^ S cm^−1^ at 25 °C [21]. In addition, chemical cross-linking is an effective way to reduce the crystalline behavior of PVDF and thus increase conductivity. Chen et al. introduced an innovative gel polymer electrolyte (GPE) by incorporating polyethylene glycol (PEG) into the crosslinking matrix of polyethylene oxide (PEO) and PVDF-HFP. This modification resulted in the formation of a crosslinked network that endows the GPE with several advantageous properties, including enhanced flexibility, elevated mechanical strength, high ionic conductivity (measured at 7.5 × 10^−4^ S cm^−1^ at room temperature), and the ability to self-heal without the need for external stimuli [18]. Cao et al. prepared porous PVDF-HFP membranes by solvent evaporation using dibutyl phthalate, polyvinylpyrrolidone, and polyethylene glycol as additives [22]. The membrane made from PVDF-HFP exhibited the highest conductivity, at 0.49 × 10^−^^3^ S/cm, and demonstrated good mechanical strength of 5.78 MPa. However, the PVDF membranes prepared using this method do not have a good balance of mechanical and electrochemical properties.

In previous studies [23,24,25,26,27,28,29], the crystallization behaviors of semi-crystalline poly(vinylidene fluoride) (PVDF) and various hydrophilic amorphous polymer blends were investigated, including polymethyl methacrylate, polyacrylonitrile, poly(ethylene glycol) methacrylate, thermoplastic polyurethane elastomer, styrene-co-acrylonitrile copolymer, and poly(methyl methacrylate)-block-poly(2-vinyl pyridine) block copolymer. Additionally, the crystallization kinetics and crystal form transitions of PVDF blended with the ionic liquid 1-hexyl-3-methylimidazolium chloride ([HMIM]Cl) were examined, and we found that [HMIM]Cl promotes the formation of the β phase of PVDF during the re-crystallization process.

Here, we report a method to enhance both the ionic conductivities and mechanical properties of GPEs based on PVDF and ionic liquids. The structures, crystallization behaviors, and properties of PVDF and [HMIM]Cl blends after solution casting and hot pressing were investigated. For comparison, PVDF-HFP/[HMIM]Cl blends were also studied. X-ray diffraction (XRD), Fourier transform infrared spectroscopy (FT-IR), and mechanical tests were employed to explore the mechanisms underlying the improvements in ionic conductivities and mechanical properties of the PVDF/[HMIM]Cl films.

## 2. Experimental Part

### 2.1. Materials

PVDF (Kynar 760, *M*_w_ = 371 kg·mol^−1^) was obtained from Arkema Inc., King of Prussia, PA, North America (USA). PVDF-HFP was obtained from Sigma Aldrich (St. Louis, MO, USA). The ionic liquid (IL) 1-hexyl-3-methylimidazolium chloride, [HMIM]Cl (≥99%), was purchased from Lanzhou Institute of Chemical Physics (Lanzhou, China). *N*,*N*-dimethylformamide (DMF) with a purity of 99% was supplied by Sinopharm Chemical Reagent Co., Ltd. (Shanghai, China). The chemical regents were used as received.

### 2.2. Film Preparation

Polymer and [HMIM]Cl films were prepared by solution casting. In total, 1.2 g PVDF or PVDF-HFP was dissolved in DMF at a concentration of 7 wt% and stirred using a magnetic stirrer for 4 h at 50 °C. Then, [HMIM]Cl with different masses were added to the PVDF solution and stirred continuously for 4 h to obtain homogeneous viscous solutions. The blending ratios are listed in Table 1. The viscous solutions were cast into glass petri dishes and evaporated using common solvents at 55 °C to obtain free-standing and semi-transparent GPE films. The solution-cast films were hot-pressed at different temperatures around the softening point under 10 MPa using a platform vulcanizing machine (China) for 10 min.

### 2.3. Characterization

#### 2.3.1. Dynamic Mechanical Thermal Analysis

Dynamic mechanical thermal analysis (DMTA) was conducted utilizing a rheometer (MCR302, Anton Paar, Graz, Austria). The sample tests were executed in torsion mode at a constant frequency of 1 Hz. The experimental temperature ranged from 30 to 200 °C, with a heating rate of 5 °C/min. All samples were prepared in a rectangular configuration measuring 10 × 6 × 0.2 mm. The storage modulus (G′) was determined as a function of temperature for each sample under uniform testing conditions. The temperature at which the G′ curves exhibited an inflection point was designated as T_s_.

#### 2.3.2. Ionic Conductivity

Ion conductivity measurements were conducted utilizing alternating current (AC) impedance spectroscopy with an electrochemical analyzer (CHI650D, Shanghai Chenhua Co., Ltd., Shanghai, China). The membrane samples had a diameter of 12 mm, and the frequency range employed was from 1 to 10^5^ Hz, with an AC perturbation voltage set at 5 mV.

#### 2.3.3. X-Ray Diffraction

The polyvinylidene fluoride (PVDF) film samples were subjected to reflection scanning at a rate of 10 degrees per minute utilizing wide-angle X-ray diffraction (WAXD) with a Shimadzu XRD-6000 instrument (Kyoto, Japan), employing Cu Kα radiation (λ = 1.542 Å) at 40 kV and 30 mA. The deconvolution of the WAXD peaks was executed using Peak Fit software (Origin 2020), which applies a mathematical function referred to as “Gaussian area”. The degree of crystallinity (*X_c_*) was calculated according to Equation (1):

(1) $$X_{c}=\frac{\sum A_{c}}{\sum A_{c}+\sum A_{am}}\times 100\%$$

where ∑*A_c_* and ∑*A_am_* are the total integral areas of the crystalline peaks and the amorphous halo from the samples, respectively.

#### 2.3.4. Differential Scanning Calorimetry (DSC)

Thermal analysis was conducted using a Q200 differential scanning calorimeter (DSC) from TA Instruments, New Castle, DE, USA. The melting behavior of the samples was examined by heating them from −70 °C to 200 °C at a rate of 10 °C/min following a cooling process. The crystallinity (*X_c_*) of the sample was determined using the equation provided below:

(2) $$X_{c}=\frac{\Delta H_{m}}{\Delta H_{m}^{*}\times \varphi }\times 100\%$$

where $\Delta H_{m}$ is the enthalpy of the blends, $\Delta H_{m}^{*}$ (104.5 J g^−1^) is PVDF with *X_c_* = 100%, and φ represents the mass fraction of PVDF in IL/PVDF blends.

#### 2.3.5. Fourier Transform Infrared Spectroscopy (FTIR)

Fourier transform infrared spectroscopy (FTIR) was performed at room temperature using a NEXUS670 spectrometer (NEXUS, Tianjin, China) in attenuated total reflection (ATR) mode. Spectra in the optical range of 600–4000 cm^−1^ were obtained by averaging 16 scans at a resolution of 4 cm^−1^.

#### 2.3.6. Mechanical Properties

Tensile tests were carried out using a universal testing machine (CMT 5254, Shenzhen SANS Testing Machine, Co., Ltd., Shenzhen, China). The initial grip separation was set to 60 mm, and film samples (2 × 5 cm) were stretched at a speed of 50 mm·s^−1^, following ISO 527 [30]. At least eight replicates of each film type were tested for two tensile properties.

## 3. Results and Discussion

In solution-cast PVDF films, PVDF exhibits spherulite structures and a β-crystal form. The distribution of crystals is uniform. Our previous study demonstrated that the addition of ionic liquids (ILs) promotes the formation of the β phase of PVDF during the recrystallization process [19]. However, PVDF will inevitably recrystallize preferentially in the direction of applied pressure when subjected to 10 MPa at varying temperatures. During this process, both the *X_c_* and crystal forms will undergo significant changes. Consequently, the distribution of ionic liquids will be reorganized during hot-pressing recrystallization.

**Figure 1:**
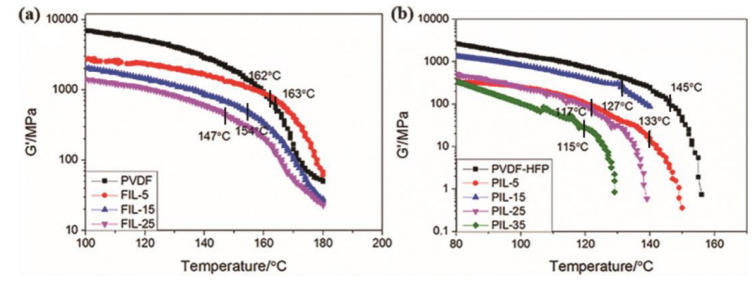
The softening point (T_s_) of (**a**) PVDF and PVDF/[HMIM]Cl blends and (**b**) PVDF-HFP and PVDF-HFP/[HMIM]Cl blends as determined from the storage modulus curve.

The selection of pressing temperature is crucial for the recrystallization process. The pressing temperature was determined based on the DMTA results of the FIL and PIL blends. Figure 1 illustrates their DMTA testing results. For both PVDF and PVDF-HFP containing [HMIM]Cl, the softening temperature (T_s_) was identified by observing the transition of the storage modulus (G′). The storage modulus G′ decreased with increasing [HMIM]Cl content, and the Ts of the FIL blends was significantly higher than that of the PIL blends. This difference is primarily due to the hexafluoropropylene in PVDF, which enhances the segmental flexibility of PVDF-HFP. Additionally, the T_s_ value exhibited a downward trend with increasing [HMIM]Cl content, albeit with some irregularities. This trend indicates that the incorporation of [HMIM]Cl facilitates segmental movement, rendering the FIL and PIL blends more flexible and resulting in a lower softening point for the blends. Consequently, we established the hot-pressing temperatures at T_su_ (under T_s_, about 90 °C), T_s_, and T_so_ (over T_s_, 150–180 °C) based on the DMTA test results to produce the hot-pressed film for further study.

### 3.1. Ionic Conductivities of the GPEs

The ionic conductivities of the FIL and PIL films were evaluated after the solution-casting and hot-pressing processes, as shown in Table 2. The σ_sc_ of FIL increases with the [HMIM]Cl content: zero, 3.02 × 10^−6^, and 3.34 × 10^−5^ S·cm^−1^, which correspond to FIL-5, FIL-15, and FIL-25, respectively. The σ_sc_ of PIL is 3.30 × 10^−6^, 1.74 × 10^−6^, 7.39 × 10^−5^ and 1.73 × 10^−4^ S·cm^−1^, which correspond to PIL-5, PIL-15, PIL-25, and PIL-35, respectively. At the same [HMIM]Cl content of 25%, the σ_sc_ of PIL-25 is nearly twice that of FIL-25 due to the presence of hexafluoropropylene in PVDF, which enhances the segmental flexibility of PVDF-HFP.

In Table 2, most of the ionic conductivities of hot-pressed (σ_T_) FIL films increase after hot pressing. For example, the ionic conductivity of FIL-5 increases from zero to 3.39 × 10^−7^ at T_su_, 3.83 × 10^−7^ at T_s_, and 1.81 × 10^−6^ S·cm^−1^ at T_so_. In most cases, as the temperature increases, the ionic conductivity of the hot-pressed FIL film also increases. Comparing σ_sc_ = 3.34 × 10^−5^ with σ_Tso_ = 4.17 × 10^−4^ S·cm^−1^ of FIL-25, the conductivity increases by a factor of 12.5 due to hot-pressing recrystallization. However, the ion conductivity of PIL changes in the opposite direction after hot pressing. In Table 2, σ_T_ generally decreases with an increase in ionic liquid content and hot-pressing temperature. For example, the ionic conductivity of PIL-35 decreases from 1.73 × 10^−4^ to 1.02 × 10^−4^ at T_su_, 2.06 × 10^−8^ at T_s_, and 1.51 × 10^−7^ S·cm^−1^ at T_so_. Only the σ_Tsu_ of the PIL-15, 1.52 × 10^−5^ S·cm^−1^, is higher than σ_sc_. Moreover, the σ_Tso_ of FIL-25, with lower IL content, has even higher ionic conductivity than the σ_sc_ of PIL-35, with higher IL content.

**Figure 2:**
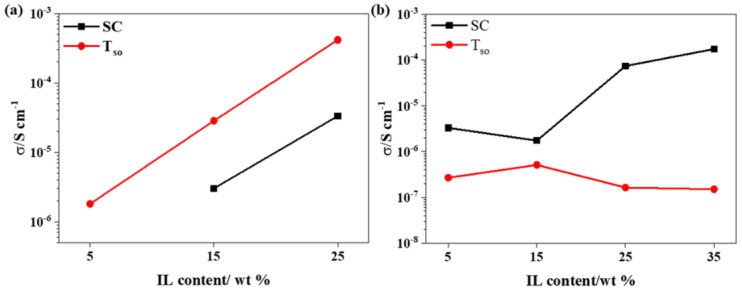
The conductivity of (**a**) PVDF/[HMIM]Cl films and (**b**) PVDF-HFP/[HMIM]Cl films with different IL content by solution casting and hot pressing at T_so_.

Figure 2 illustrates the comparison of σ_sc_ and σ_Tso_ for the solution-cast and hot-pressed FIL and PIL films. In Figure 2a, the σ_sc_ of FIL increases linearly with the [HMIM]Cl content, and the PIL shows a similar increasing trend, except for PIL-15. Combining Table 2 with Figure 2, the conductivity of the hot-pressed FIL film is significantly superior to that of the PIL film. The flexibility of the polymer chains is enhanced, resulting in more favorable ion transport [31,32]. Increasing the temperature and adding an appropriate IL content would enhance the flexibility of the molecular chain, thereby improving ion mobility. However, in the case of PVDF-HFP, which contains hexafluoropropylene, the addition of [HMIM]Cl results in an excessive amount of plasticizer in the PIL. Under hot-pressing conditions, this excess plasticizer can hinder the movement of the chain segments and restrict particle migration, leading to irregular fluctuations in the conductivity of the hot-pressed PIL film.

In conjunction with the aforementioned conductivity, we selected solution-cast films of FIL-25 and PIL-25 (designated as FIL-25-S and PIL-25-S, respectively) as well as hot-pressed films at Ts (designated as FIL-25-H and PIL-25-H, respectively) to analyze the mechanical properties of the GPE film. The results are presented in Table 3.

**Figure 3:**
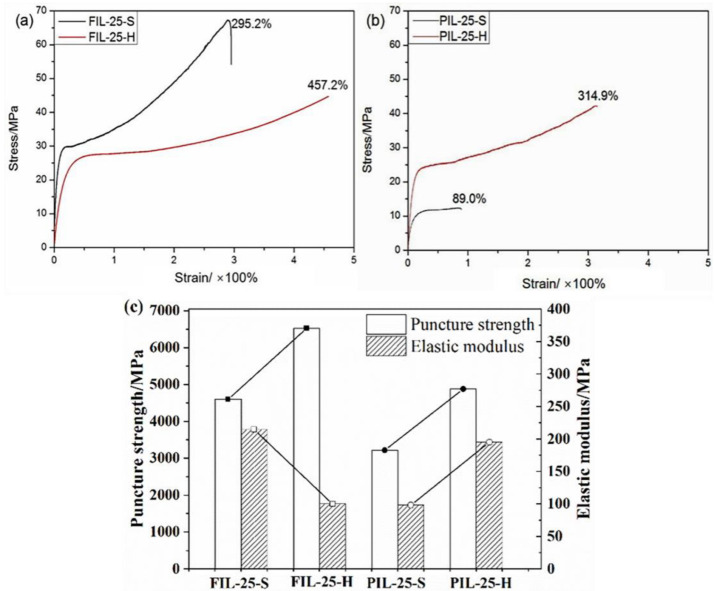
Tensile stress–strain curves of (**a**) FIL-25 and (**b**) PIL-25, and (**c**) the puncture strength and elastic modulus of FIL-25 and PIL-25.

It can be observed that the tensile strength of FIL-25-S, at 67.25 MPa, is higher than that of FIL-25-H, at 46.83 MPa. The elongation at break of 295.2% is lower than that of FIL-25-H, which is 457.2%. Compared to PIL-25-S, PIL-25-H exhibits a significant increase in σ_t_ (42.33 MPa) and ε_b_ (314.9%). Figure 3c illustrates the puncture strength and elastic modulus of the blended film. The puncture strength of FIL-25-H is 6527.3 MPa, which is nearly 42% higher than the 4604.6 MPa of FIL-25-S. However, its elastic modulus of 100.1 MPa decreased by nearly 53.3%. This reduction is primarily due to the addition of IL and the hot-pressing method, which enhances the flexibility of the segment and improves the material’s toughness. In comparison to PIL-25-S, the puncture strength of PIL-25-H increased by nearly 51.5%. Additionally, its elastic modulus of 195.2 MPa also rose by approximately 98.9%.

Through the results presented, hot-pressing recrystallization significantly enhanced the ion conductivity, puncture strength, and elongation at break of solution-cast FIL films, while reducing their tensile strength and elastic modulus. In contrast, for the PIL blend film, the ion conductivity of the solution-cast film decreased substantially after hot-pressing; however, its puncture strength, elongation at break, tensile strength, and elastic modulus all showed improvement. As is well known [33,34,35,36], the ion conductivity of GPE films and their mechanical strength often exhibit contradictory behaviors. The recrystallization processes of FIL and PIL blend films, both before and after hot pressing, present intriguing opportunities for further research [37].

### 3.2. Crystallinity

**Figure 4:**
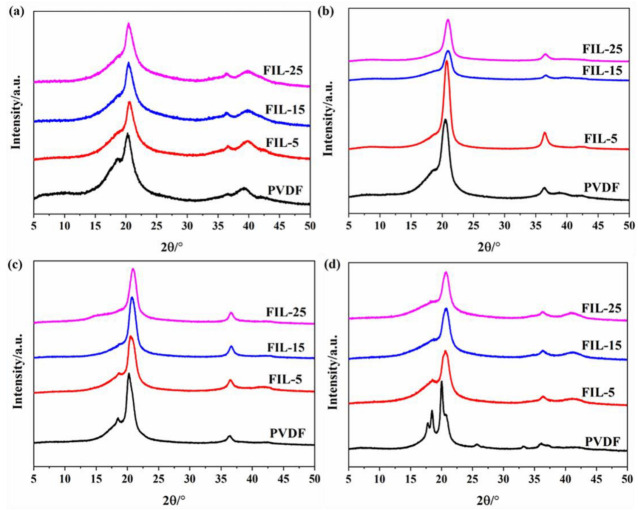
The XRD patterns of pure PVDF and PVDF/IL blends: (**a**) solution-cast films, (**b**) hot-pressed films (under T_s_), (**c**) hot-pressed films (at T_s_), and (**d**) hot-pressed films (over T_s_).

**Figure 5:**
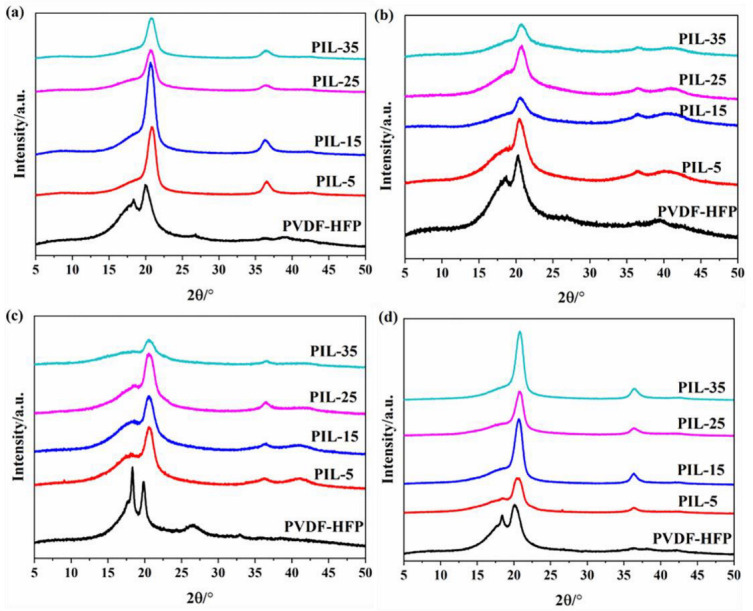
The XRD patterns of pure PVDF-HFP and PVDF-HFP/IL blends: (**a**) solution-cast films, (**b**) hot-pressing films (under T_s_), (**c**) hot-pressed films (at T_s_), and (**d**) hot-pressed films (over T_s_).

To investigate the crystal phase and degree of crystallinity of PVDF and PVDF-HFP, wide-angle X-ray diffraction (WAXD) experiments were conducted. The X-ray diffractograms of the PVDF films are shown in Figure 4 and Figure 5.

In Figure 4, the WAXD profiles for the solution-cast film of pure PVDF and FIL blends exhibit one prominent peak corresponding to the crystalline phase at a diffraction angle of 20.5°, along with two weaker peaks at 36.6° and 39.8°. These peaks are associated with the reflections of the (200), (020), and (400) planes, respectively, which are characteristic of the orthorhombic unit cell of PVDF in its β crystalline phase [28,38,39,40]. Therefore, the β phase is identified as the predominant crystalline phase in the solution-cast films.

As shown in Figure 4, the WAXD curves of pure PVDF are similar to those of the solution-cast film when the hot-pressing temperature is at T_su_ and T_s_, indicating that the β phase remains dominant. However, the XRD curves of pure PVDF change significantly when the hot-pressing temperature reaches T_so_. The curve reveals four strong peaks of the crystalline phase at diffraction angles of 17.8°, 18.5°, 20.2°, and 26.8°, indicating that the α phase is the predominant crystalline phase [24,40,41,42]. This situation indicates that the crystal structure of pure PVDF was altered during the hot-pressing process as the temperature increased. Meanwhile, the β phase continues to dominate in the FIL blends throughout the hot pressing.

The crystalline phase evolution of the PVDF-HFP samples was systematically investigated using WAXD analysis. For pure PVDF-HFP (Figure 5a), the characteristic diffraction peaks observed at 18.5° and 19.9° correspond to the (020) and (110) reflections of the α-phase crystal structure, respectively. Notably, solution-cast PIL-blended films exhibit distinct crystalline features, with a prominent diffraction peak at 20.5° accompanied by two secondary peaks at 36.6° and 39.8°. These diffraction patterns align with the (200), (020), and (400) crystallographic planes of the orthorhombic β phase, demonstrating a significant phase transformation from the non-polar α phase to the electroactive β phase upon ionic liquid incorporation. This β-phase predominance (approximately 82% crystallinity based on peak deconvolution analysis) can be attributed to the strong ion–dipole interactions between PVDF-HFP chains and ionic liquid molecules, which promote all-trans (TTTT) chain conformation during solution crystallization.

Remarkably, the WAXD profiles of hot-pressed films (Figure 5b–d) maintain diffraction patterns similar to those of their solution-cast counterparts, with β-phase characteristic peaks remaining prominent despite thermal processing. This thermal stability of the β-phase crystalline structure suggests that the ionic liquid acts as both a plasticizer and structural template during the melt-processing stage, effectively suppressing the thermally induced reversion to the α phase that typically occurs above 160 °C in neat PVDF systems. The persistence of β-phase crystallinity under hot-pressing conditions (180–200 °C, depending on IL content) highlights the critical role of ionic interactions in stabilizing the polar phase, which is particularly advantageous for maintaining piezoelectric performance in melt-processed devices. Comparative analysis of peak-broadening effects reveals slight reductions in crystallite size after hot pressing, possibly due to partial chain disordering during thermal treatment, though the fundamental crystal structure remains intact [31,38,43].

**Figure 6:**
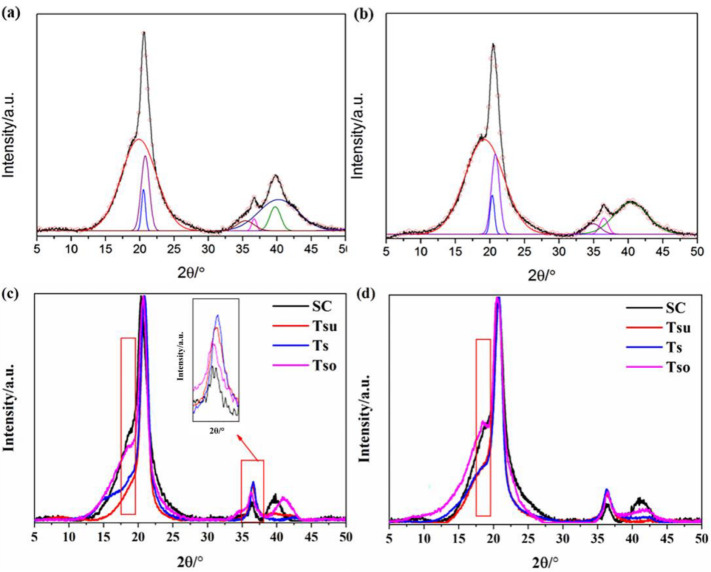
XRD patterns and their curve deconvolution of the solution-cast film. (**a**) FIL-5 and (**b**) PIL-5; XRD baseline correction maps for (**c**) FIL-25 and (**d**) PIL-25.

The crystallinity evolution and electrical performance correlations were quantitatively analyzed through the deconvolution of WAXD patterns (Figure 6). As summarized in Table 4, pure PVDF solution-cast films exhibited a crystallinity degree (*X_c_*) of 32.2%, consistent with typical α-phase-dominated systems [33]. Remarkably, [HMIM]Cl incorporation induced significant structural modifications—the Xc values progressively decreased from 19.1% (FIL-5) to 18.4% (FIL-25), as evidenced by the characteristic peak broadening in the reverse roll integral curves (Figure 6a,b). This crystallinity suppression (∼40% reduction vs. pure PVDF) confirms strong polymer–ion interactions that disrupt chain packing efficiency, likely through cation coordination with PVDF’s CF2 groups and chloride anion hydrogen bonding.

Thermal processing introduced complex crystallization behavior: while pure PVDF showed the expected Xc enhancement from 27.8% to 37.8% with elevated hot-pressing temperatures (attributed to improved chain mobility [44]), FIL-5 displayed inverse trends, with Xc decreasing from 48.7% to 39.8%. However, FIL-15/25 systems exhibited opposing trends, where Xc reduction (∼15%) coincided with conductivity deterioration—a dichotomy highlighting competing mechanisms: (1) IL-induced amorphous domain expansion facilitating ion transport vs. (2) excessive phase segregation disrupting conductive pathways at higher IL loadings.

Notably, hot-pressed FIL-15-H/25-H demonstrated 23–35% higher conductivity than the solution-cast counterparts despite 12–18% greater *X_c_* values—contradicting conventional crystallinity–conductivity inverse relationships. This paradox suggests that (1) preferential β-phase alignment during thermal processing enhances dipolar polarization despite higher crystallinity, (2) nanoscale ionic clustering within amorphous regions creates percolation networks unaffected by crystalline fraction, and (3) thermal annealing eliminates solution-casting defects that hinder charge transport.

Additionally, pure PVDF-HFP exhibits an *X_c_* of 27.1%. For neat PVDF-HFP, increasing the hot-pressing temperature results in a systematic reduction of *X_c_* from 30.0% to 25.3%. In PIL blends, solution-cast films exhibit a decrease in *X_c_* from 16.1% to 14.2% with increasing [HMIM]Cl content (5.0–35.0 wt%). Notably, hot-pressing treatment improves the crystallinity of all PIL blends, yet results in reduced ionic conductivity compared to as-cast samples. For PIL-5 and PIL-15 compositions, hot-pressed films show temperature-dependent decreases in *X_c_*, contradicting the conductivity trends. Conversely, PIL-25 and PIL-35 achieve maximum *X_c_* values of 36.3% and 49.3%, respectively, with PIL-35 demonstrating the highest crystallinity across all thermal treatments. These observations highlight that factors beyond crystallinity play a significant role in governing the ionic conductivity of PIL-modified PVDF-HFP electrolytes [36,45,46].

Based on the analysis of *X_c_* presented above, the FIL-25 and PIL-25 samples were selected for further discussion. Figure 6c,d presents the baseline-corrected XRD patterns for FIL-25 and PIL-25 electrolytes, respectively, designed to elucidate the relationship between polymer crystallinity and ionic conductivity [36]. Specifically, for FIL-25, the diffraction peak at 18.6° in the hot-pressed film remained essentially unchanged compared to its solution-cast counterpart. Conversely, the peak at 36.6° exhibited significant intensity enhancement under thermal treatment.

Under different thermal treatment conditions, hot pressing at T_su_ and T_s_ temperatures systematically increases the diffraction peak intensity of the films with rising temperature, whereas treatment at Tso demonstrates a decreasing trend. Specifically, the peak at 39.9° completely vanishes in samples processed at T_su_ and T_s_, while it exhibits a significant shift to 41.1° (higher 2θ values) under T_so_ conditions. This observation suggests that PVDF undergoes structural rearrangement during the hot-pressing recrystallization process, potentially leading to an anisotropic orientation of crystalline domains along the pressure direction (perpendicular to the film surface), which may facilitate ion transport pathways [23,39]. For PIL-25 electrolytes, hot-pressed films exhibit a weak diffraction peak at 18.6° compared to their solution-cast counterparts. The peak at 36.6° only shows intensity enhancement when processed at T_so_, reaching its maximum intensity at T_s_. Concurrently, the peak at 41.1° transforms into a broad hump, indicating that PVDF-HFP undergoes both crystal phase transformation and anisotropic formation during thermal treatment. However, these structural changes are not the primary determinants of ionic conductivity in electrolyte systems [47,48].

**Figure 7:**
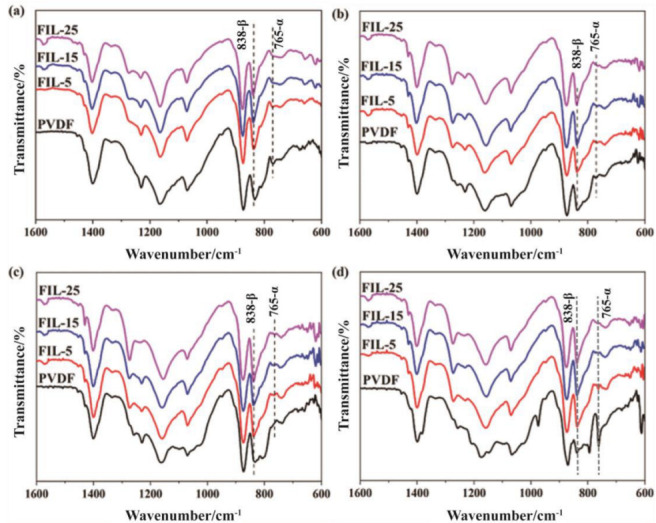
The FT-IR patterns of pure PVDF and PVDF/IL films: (**a**) solution-cast films, (**b**) hot-pressed films (under T_s_), (**c**) hot-pressed films (at T_s_), and (**d**) hot-pressed films (over T_s_).

**Figure 8:**
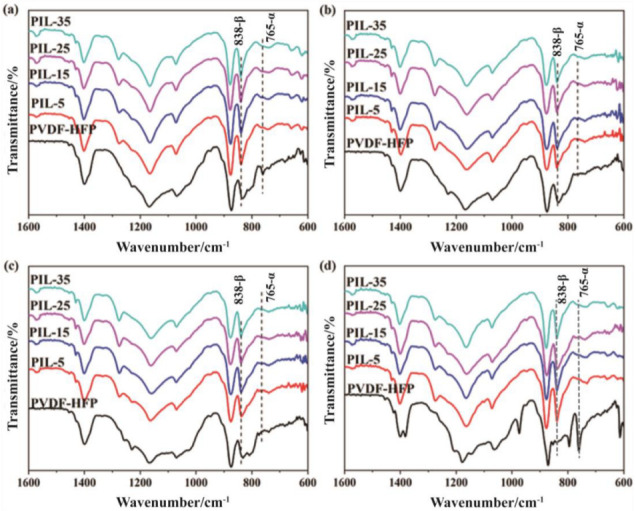
The FT-IR patterns of pure PVDF-HFP and PVDF-HFP/IL films: (**a**) solution-cast films, (**b**) hot-pressed films (under T_s_), (**c**) hot-pressed films (at T_s_), and (**d**) hot-pressed films (over T_s_).

The crystalline phases of the PVDF and PVDF-HFP solution-cast films and hot-pressed films containing varying amounts of IL were identified at room temperature (RT) using Fourier transform infrared spectroscopy (FTIR), as shown in Figure 7 and Figure 8, respectively. In the presence of IL within the PVDF or PVDF-HFP matrix, the β phase of the polymer predominates, characterized by absorption bands at 838 cm^−^^1^ and 1279 cm^−^^1^. However, the weak absorption bands observed at 765 cm^−^^1^, 1070 cm^−^^1^, and 1423 cm^−^^1^ indicate the presence of low α-phase content.

The relative fraction of the β phase in a sample containing just α and β phase is

(3) $$F(\beta )=\frac{A_{\beta }}{A_{\beta }+(K_{\beta }/K_{\alpha })A_{\alpha }}$$

where F(β) represents the β-phase content; A_α_ and A_β_ are the absorbance at 765 and 838 cm^−1^; K_α_ and K_β_ are the absorption coefficients at the respective wavenumber, with values of 6.1 × 10^4^ and 7.7 × 10^4^ cm^2^·mol^−1^, respectively. According to Equation (3), the calculated β-phase content for PVDF is listed in Table 5.

The β-phase content of solution-cast films is 78.0%, 90.3%, 92.5%, and 93.6%, corresponding to pure PVDF, FIL-5, FIL-15, and FIL-25, respectively. Clearly, the β-phase content of solution-cast films increases with the addition of [HMIM]Cl, indicating that the inclusion of [HMIM]Cl promotes the crystallization of the β phase in PVDF. For pure PVDF, the β-phase content of hot-pressed films is 91.1%, 98.8%, and 32.3%, corresponding to different hot-pressing temperatures. This demonstrates that the hot-pressing temperature (T_so_) is not conducive to the formation of the β phase. Furthermore, the β-phase content for hot-pressed films is 98.6%, 97.8%, and 94.8% for FIL-5 at varying temperatures, respectively. It is evident that the β-phase content in hot-pressed films decreases as the hot-pressing temperature increases. When the [HMIM]Cl content in PVDF decreases, the interaction between the ions and the polymer chains strengthens, inhibiting the formation of the β phase as the hot-pressing temperature rises.

The β phase content of hot-pressed films of FIL-15 and FIL-25 is similar and significantly higher than that of FIL-5 under the same conditions. This indicates that as the [HMIM]Cl content in PVDF increases, the interaction between the ions and the polymer chains intensifies, resulting in an increase in the β-phase content. Furthermore, as the [HMIM]Cl content continues to rise, the ion pairs become saturated, leading to a gradual stabilization of the interaction, which causes the β-phase content in FIL-15 and FIL-25 to converge. The β-phase content of hot-pressed film is greater than that of solution-cast film in the FIL blends, suggesting that during the hot-pressing process, the PVDF chains undergo rearrangement, and the interaction between the ions and the chains may facilitate the formation of the β phase. Considering the changes in conductivity between solution-cast films and hot-pressed films, the β phase positively influences the conductivity of FIL blends.

The β-phase content values for solution-cast films are 71.5%, 96.4%, 96.5%, 96.3%, and 96.8%, corresponding to pure PVDF-HFP, PIL-5, PIL-15, PIL-25, and PIL-35, respectively. This suggests that [HMIM]Cl in PVDF-HFP influences the formation of the β phase, but its effect appears to be independent of the [HMIM]Cl content. For the hot-pressed film of PIL blends, the variation in β-phase content is particularly noteworthy. At the hot-pressing temperature of T_su_, the F_c_T_su_(β) increases from 98.0% to 98.6% as the [HMIM]Cl content rises from 5.0 wt% to 25.0 wt%. However, when the [HMIM]Cl content is further increased to 35.0 wt%, the F_c_T_su_(β) decreases back to 98.0%. At the hot-pressing temperature of T_s_, the F_c_T_s_(β) increases from 97.8% to 98.8% as the [HMIM]Cl content increases from 5.0 wt% to 15.0 wt%. With a further increase in [HMIM]Cl content, the F_c_T_s_(β) subsequently decreases from 98.8% to 97.9%. When the hot-pressing temperature is at Tso, the F_c_T_so_(β) decreases from 99.3% to 97.3% as the [HMIM]Cl content increases from 5.0 wt% to 35.0 wt%.

When the hot-pressing temperature is at Tsu, the molecular chains and segments are unable to move during the process, which prevents the ion pairs from moving freely. As a result, when the [HMIM]Cl content ranges from 5.0 to 25.0 wt%, the ion pairs can further disperse into the amorphous region, promoting the crystallization of the β phase during the rearrangement of the PVDF chains. However, when the [HMIM]Cl content increases to 35.0 wt%, the ion pairs become saturated, causing the remaining pairs to cluster together. This clustering hinders the rearrangement of the PVDF chains, thereby inhibiting the formation of the β phase. When the hot-pressing temperature is at Ts, only the segments can begin to move during the process, allowing the ion pairs to move freely within a small range. Consequently, when the [HMIM]Cl content increases to 25.0 wt%, some of the ion pairs may already cluster together, inhibiting the formation of the β phase. When the hot-pressing temperature reaches Tso, both the molecular chains and segments can move during the process, enabling the ion pairs to move freely and aggregate more easily. Therefore, the F_c_T_so_(β) decreases as the [HMIM]Cl content in PIL blends increases. Considering the changes in conductivity of the solution-cast film and the hot-pressed film, the β phase may have a minimal effect on the conductivity of PIL blends.

## 4. Conclusions

We significantly improved the ion conductivity of the original solution-cast PVDF/[HMIM]Cl films through hot-pressing recrystallization. For FIL-25, the ion conductivity increased by 12.5 times, rising from 3.34 × 10^−5^ to 4.17 × 10^−4^ S·cm^−1^ in the thickness direction. Furthermore, the puncture strength of FIL-25-H also increased by 42% compared to FIL-25-S after hot pressing. Conversely, in-plane mechanical properties revealed an increase in elongation at break from 295.2% to 457.2%, accompanied by reductions in tensile strength (67.25 MPa → 46.83 MPa) and elastic modulus (214.5 MPa → 100.1 MPa), indicative of oriented recrystallization. For PIL, the conductivity of the hot-pressed film was significantly lower than that of the solution-cast film, but its mechanical strength was greatly enhanced due to the increase in polymer crystallinity (*X_c_*). The hot-pressing-induced crystalline structures in PVDF-based gel polymer electrolytes (GPEs) simultaneously benefited both ion transport pathways and puncture resistance.

## Figures and Tables

**Table 1:** The mixing ratio of polymers with ionic liquid and abbreviation.

	Polymer/wt%	[HMIM]Cl/wt%	Abbreviation
PVDF	95	5	FIL-5
	85	15	FIL-15
	75	25	FIL-25
PVDF-HFP	95	5	PIL-5
	85	15	PIL-15
	75	25	PIL-25
	65	35	PIL-35

**Table 2:** The ion conductivity of the solution-cast and hot-pressed FIL and PIL films.

Sample	σ_SC_ */S·cm^−1^	σ_Tsu_ */S·cm^−1^	σ_Ts_ */S·cm^−1^	σ_Tso_ */S·cm^−1^
FIL-5	-	3.39 × 10^−7^	3.83 × 10^−7^	1.81 × 10^−6^
FIL-15	3.02 × 10^−6^	1.85 × 10^−5^	6.75 × 10^−6^	2.85 × 10^−5^
FIL-25	3.34 × 10^−5^	7.49 × 10^−5^	9.39 × 10^−5^	4.17 × 10^−4^
PIL-5	3.30 × 10^−6^	3.91 × 10^−8^	1.73 × 10^−7^	2.69 × 10^−7^
PIL-15	1.74 × 10^−6^	1.52 × 10^−5^	1.49 × 10^−7^	5.12 × 10^−7^
PIL-25	7.39 × 10^−5^	4.21 × 10^−6^	6.07 × 10^−7^	1.64 × 10^−7^
PIL-35	1.73 × 10^−4^	1.02 × 10^−4^	2.06 × 10^−8^	1.51 × 10^−7^

* σ_SC_: conductivity of solution-cast film; σ_Tsu_: conductivity of hot-pressed film at T_su_; σ_Ts_: conductivity of hot-pressed film at T_s_; σ_Tso_: conductivity of hot-pressed film at T_so_.

**Table 3:** The mechanical properties of the polymers and IL blends.

Sample	σ_PS_/MPa	σ_t_/MPa	ε_b_/%	E/MPa
FIL-25-S	4604.6	67.25	295.2	214.5
FIL-25-H	6527.3	46.83	457.2	100.1
PIL-25-S	3220.4	12.45	89.0	98.1
PIL-25-H	4880.3	42.33	314.9	195.2

σ_PS_: puncture strength; σ_t_: tensile strength; ε_b_: elongation at break; E: elastic modulus.

**Table 4:** The degree of crystallinity of the FIL and PIL blends as determined by XRD.

Sample	X_Csc_/%	X_CTsu_/%	X_CTs_/%	X_CTso_/%
PVDF	32.2	27.8	33.4	37.8
FIL-5	19.1	48.7	43.8	39.8
FIL-15	19.1	33.9	45.2	37.3
FIL-25	18.4	42.0	35.0	32.0
PVDF-HFP	27.1	30.0	25.3	25.4
PIL-5	16.1	42.8	28.3	28.6
PIL-15	15.0	41.6	38.6	28.3
PIL-25	19.3	32.1	36.3	28.4
PIL-35	14.2	34.2	49.3	17.1

X_csc_: X_c_ of solution-cast film; Xc_Tsu_: X_c_ of hot-pressed film at T_su_; Xc_Ts_: X_c_ of hot-pressed film at T_s_; Xc_Tso_: X_c_ of hot-pressed film at T_so_.

**Table 5:** The β-phase content of the FIL and PIL blends as determined by FTIR.

Sample	F_CSC_(β)/%	F_CTsu_(β)/%	F_CTs_(β)/%	F_CTso_(β)/%
PVDF	78.0	91.1	98.8	32.3
FIL-5	90.3	98.6	97.8	94.8
FIL-15	92.5	98.7	98.4	98.8
FIL-25	93.6	98.7	98.4	99.1
PVDF-HFP	71.5	99.0	92.9	3.1
PIL-5	96.4	98.0	97.8	99.3
PIL-15	96.5	98.3	98.8	98.8
PIL-25	96.3	98.6	98.4	98.6
PIL-35	96.8	98.0	97.9	97.3

F_CSC_: β-phase content of solution-cast films, F_CTsu_: β-phase content of hot-pressed film at T_su_, F_CTs_: β-phase content of hot-pressed film (at T_s_), Fc_Tso_: β-phase content of hot-pressed film at T_so_.

## Data Availability

The original contributions presented in the study are included in the article, further inquiries can be directed to the corresponding author.
